# Simulating Signal Aberration and Ranging Error for Ultrasonic Indoor Positioning

**DOI:** 10.3390/s20123548

**Published:** 2020-06-23

**Authors:** Riccardo Carotenuto, Massimo Merenda, Demetrio Iero, Francesco G. Della Corte

**Affiliations:** 1 DIIES Department, University Mediterranea of Reggio Calabria, 89126 Reggio Calabria, Italy; r.carotenuto@unirc.it (R.C.); demetrio.iero@unirc.it (D.I.); francesco.dellacorte@unirc.it (F.G.D.C.); 2 HWA srl, Spin-off University Mediterranea of Reggio Calabria, Via R. Campi II tr. 135, 89126 Reggio Calabria, Italy

**Keywords:** acoustic diffraction, acoustic signal aberration, cross-correlation aberration, ultrasonic ranging

## Abstract

Increasing efforts toward the development of positioning techniques testify the growing interest for indoor position-based applications and services. Many applications require accurate indoor positioning or tracking of people and assets, and some market sectors are starting a rapid growth of products based on these technologies. Ultrasonic systems have already been demonstrating their effectiveness and to possess the desired positioning accuracy and refresh rates. In this work, it is shown that a typical signal used in ultrasonic positioning systems to estimate the range between the target and reference points—namely, the linear chirp—due to the effects of acoustic diffraction, in some cases, undergoes a shape aberration, depending on the shape and size of the transducer and on the angle under which the transducer is seen by the receiver. In the presence of such signal shape aberrations, even one of the most robust ranging techniques, which is based on cross-correlation, provides results affected by a much greater error than expected. Numerical simulations are carried out for a typical ultrasonic chirp, ultrasonic emitter, and range technique based on cross-correlation and for a typical office room, obtained using the academic acoustic simulation software Field II. Spatial distributions of the ranging error are provided, clearly showing the favorable low error regions. The work demonstrates that particular attention must be paid to the design of the acoustic section of the ultrasonic positioning systems, considering both the shape and size of the ultrasonic emitters and the shape of the acoustic signal used.

## 1. Introduction

Augmented reality (AR) and many other applications based on positioning are emerging technologies that need indoor positioning technology. Mall navigation, path finding in large hospitals or airports, the automatic guidance for unmanned cleaning and maintenance vehicles, surveillance systems, and others require positioning systems capable of operating inside buildings with high positioning accuracy [1,2,3,4,5]. Many accurate positioning systems use trilateration, a technique that has proven to work well indoors. The trilateration (multilateration) positioning technique requires three (many) range measurements between reference emitters and a sensor to be located. Distances and spatial positions with a high degree of precision at a relatively low cost can be provided by systems based on ultrasonic waves [6,7,8,9,10]. The most used ranging technique involves the estimation of the time of arrival (TOA) of a suitable ultrasonic signal. Typically, the TOA is estimated by finding in the received signal or in the postprocessed signal a specific that is easy to identify upon arrival. In some cases, the arrival time of the maximum peak of the envelope of an ultrasonic pulse is used. However, despite the simplicity of this technique, it is highly subject to acoustic disturbances, with errors in the order of wavelengths (centimeters) even in the presence of a high signal-to-noise ratio (SNR) [11,12,13,14,15,16].

Methods based on cross-correlation estimate the TOA with high accuracy and, in general, good acoustical noise immunity [13,14,15,16]. Digital cross-correlation techniques properly sample and analog-to-digital convert the received acoustical signal to obtain the array C = S ★ T, where S is the numerical array of received signal samples, T is the digital reference signal, previously stored in the sensor processor memory as a numerical array of samples, and ★ denotes the cross-correlation operator. The best aligning of S and T in time is revealed by the peak of the cross-correlation. The inter-signals displacement, or lag τ, corresponding to the cross-correlation peak (i.e., τ_MAX_), is proportional to the TOA [17,18].

The distance measurement accuracy of the order of the current space sampling, which is the distance covered by the ultrasound during the time sample interval and that can be made much smaller than the ultrasonic wavelength, is achieved by estimating the TOA through the cross-correlation peak. In addition, the cross-correlation peak is easily detectable when the signal T is a chirp. In practical systems, it is possible to achieve a range resolution up to the order of one-tenth of the wavelength used. For example, in [16], a range resolution of ± 1.2 mm was experimentally achieved using a 15–40-kHz chirp, with a wavelength range 22.86–8.57 mm, considering a sampling frequency of 1 MHz and, thus, space sampling of 0.34 mm with a sound speed of 343 m/s. When the signal and noise are uncorrelated, cross-correlation significantly increases the SNR. It is a known drawback that, in some cases, the cross correlation peak associated with the chirp that travels along the direct path (or line of sight, LOS) is lower than the signals coming from the reflection paths. In the presence of reflections, a number of signals from indirect paths combine to produce a peak higher than that of the direct path signal.

The acoustic field generated by acoustic transducers according to the shape and aperture of the transducer has been widely studied in the past considering impulsive or continuous sinusoidal wave signals. The closed-form solution of the generated acoustic field made it possible to derive simple approximate formulas to calculate the emission angle as a function of the wavelength, aperture, and distance from the emitter, the best known of which apply to circular apertures. In the far field, the emission cone semi-angle ϑ is approximately described by the well-known relationship [19]

(1) $$\sin\vartheta =1.22\frac{\lambda }{D}$$

where λ is the emitted wavelength and D the diameter, or aperture, of the circular planar transducer.

Furthermore, it is well-known that, to obtain good results, the receiver must always operate within the emission cone of the emitter [20]. At present, no equivalent formulas are known in the case of any signals, such as chirp.

To design a positioning system based on ultrasonic signals, tools to evaluate the spatial coverage of each transducer in terms of quality (amplitude and level of deformation) of the received signal are needed. For any signals, there are no simplified formulas that give a reliable indication; therefore, the usage of numerical tools is mandatory.

The use tout court of finite element analysis (FEA), which is a very powerful general use tool, seems excessive for the design of positioning systems and definitively not practical from the computational point of view. In fact, for the systems under investigation, large spatial regions of several cubic meters and time windows of tens of milliseconds should be considered (see, e.g., [9,10,13]).

From these premises, it was, therefore, decided to use a powerful numerical tool, the academic Field II software [21], for the analysis of ultrasound positioning systems. Field II was developed and is currently very well-known for the simulation of complete ultrasonic imaging systems. A transducer for ultrasonic signals both for transmission and reception, and the formation process of images in the field of medical ultrasounds, are simulated. However, Field II has the numerical characteristics that make it a valid tool also in the field of ultrasonic positioning.

Among many other capabilities, which however fall outside the scope of this work, this tool is able to calculate the acoustic pressure field at any point in the space for transducers and signals of any shape, taking into account the attenuation properties of the propagation medium. In other words, Field II allows to modify in any way the transducer shape and size, and the signal applied to the transducer, to evaluate their effects in a trial and error design cycle, if necessary.

In this work, using Field II, the effectiveness of cross-correlation-based ranging techniques using a chirp signal when the diameter of the circular plane transducer used as ultrasonic emitter is changed is shown.

This work will show that, considering a chirp signal outside a certain emission cone generated by the transducer, the usual ranging technique introduces a significant error in calculating the emitter-receiver distance.

In perspective, the main advantages of the proposed approach are the possibility of examining the acoustic field over time and space at each point of the region of interest as a function of the aperture and of the type of signal emitted (e.g., of its bandwidth or shape) and the ability to easily test each algorithm dedicated to estimating the TOA in the various positions and operating situations.

This paper is structured as follows. Section 2 describes the proposed simulation setup, while Section 3 shows the simulation results, and Section 4, the discussion. Section 5 draws the paper’s conclusions.

## 2. Field II and Simulation Setup

In this section, the operating principle of the Field II simulator is briefly outlined, and the simulation configuration is described in detail.

The acoustic field simulator Field II [22] employs the concept of spatial impulse responses [23,24,25]. The ultrasound field for both the pulsed and continuous wave cases is found using the linear systems theory. The spatial impulse response gives the emitted ultrasound field at a specific point in space as a function of time, when the transducer is excited by a Dirac delta function. In a second step, the field generated by any kind of excitation is found by convolving the spatial impulse response with the excitation function. Since the linear systems theory is used, any excitation can be considered. The impulse response is a function of the position relative to the transducer, hence the name spatial impulse response of the technique [26].

Briefly, the transducer surface is divided into small rectangles, introducing a transducer surface and field approximations that are as much smaller as the elements into which the transducer surface is divided are smaller. The approximation is reduced by using small rectangles, where the distance to the field point is large compared to the size of the rectangles. Typically, the element size is much smaller than the wavelength of the signal to be simulated. Each of the rectangular elements is considered a rectangular piston, of which the exact solution for the impulsive response is known [25]. A spherical wave is emitted by each of the small elements, and the impulsive responses due to each element are added together at each desired field point [26].

**Figure 1:**
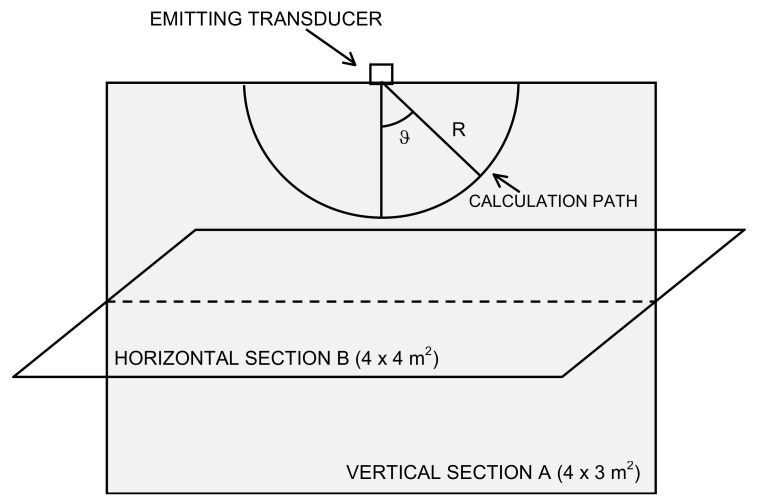
Simulation setup: the calculation path of the cross-correlation results shown in Figures 3–5; the vertical section A of the typical 4 m × 4 m × 3 m room along which the results displayed in Figure 6 are computed; the horizontal section B where the results displayed in Figure 7 are computed.

In the simulations that follow, the aim is to examine the acoustic field and the effectiveness of an established ranging technique based on the correlation in a typical 4 × 4 × 3 m^3^ room [27]. In particular, the simulation results will be examined on a grid of points belonging to a vertical section (see Section A of the room volume, Figure 1) and on a horizontal section halfway between the floor and the ceiling (see Section B of the room volume, Figure 1). The transducer is a circular planar and is placed in the center of the ceiling, in position *x* = 0, *y* = 0, and *z* = 0, and emits towards the floor of the room.

The transducer is immersed in the air, and a linearized air absorption (slope 39.3 dB/m·MHz, constant term −0.262 dB/m, i.e., about 0.917 dB/m @ 20 kHz and 1.703 dB/m @ 50 kHz) has been assumed around 40 kHz, corresponding to a transducer central frequency of 40 kHz at a temperature of 20 °C, a pressure of 1 atm, and a relative humidity of 55% [28,29].

The emitted and received signals at all points in the space depends on the shape and size of the emission surface (i.e., on the aperture D) of the transducer. In this work, a circular plane transducer was considered, with acoustic properties similar to those of the most commonly used transducers for positioning applications—for example, the typical piezoelectric transducer Murata MA40S4S (D = 9.9 mm) or Pro-wave 328ST/R160 (D = 13.1 mm) [30,31].

**Figure 2:**
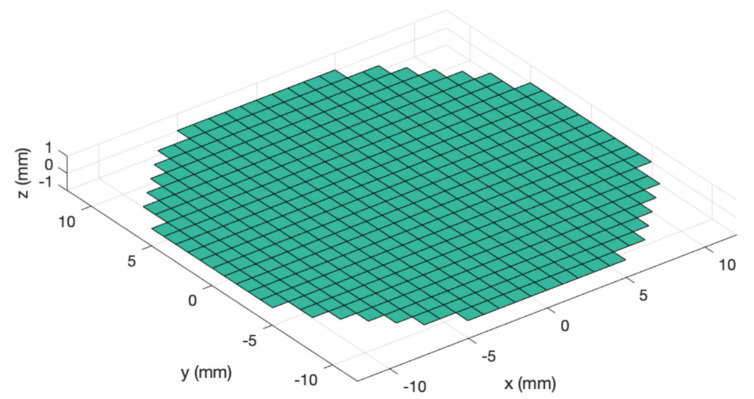
Example of a defined emitting transducer: circular and planar piston transducer with a diameter of D = 25 mm divided into square mathematical elements.

The emission disk is divided into a certain number of rectangles. In particular, square elements with sides 0.125 mm by 0.125 mm were used for all the simulations that follow. The transducer element size chosen in this work is a good compromise between the accuracy of the solution and computational resources engaged in the simulation. In Figure 2, for displaying purposes, in order to visualize the single elements, the dimension of the mathematical elements is 1 mm by 1 mm.

The signal used for the simulations is a linear chirp with a bandwidth of 30-50 kHz and 5.12-ms duration [10,27].

For simulation purposes, the signal was sampled at f_S_ = 1 MHz. The acoustic field was calculated in a set of points in the space for the duration of a time window compatible with the complete reception of the signal itself [26]. Once the simulation was completed, for each point of the space considered in the simulation, the behavior over time of the acoustic pressure generated by the complete excitation signal was obtained. This allowed any subsequent evaluation and postprocessing of the signal to be obtained. For example, the peak pressure and the total signal strength at each point can be calculated. Subsequently, an ideal receiver was assumed that linearly transduced the pressure signal into an electrical signal, downstream of a suitable sampling and numerical quantization, so that the cross-correlation vector C could be calculated.

## 3. Simulations Results

All the following simulations were performed for a set of acoustical apertures D = {25 mm, 20 mm, 15 mm, 13.1 mm, 8.5 mm, 6 mm}. The simulation includes acoustic diffractive phenomena, with the possibility of simulating transducers of every shape and every emitted signal. Finally, it is possible to test every ranging or positioning technique one intends to apply.

**Figure 3:**
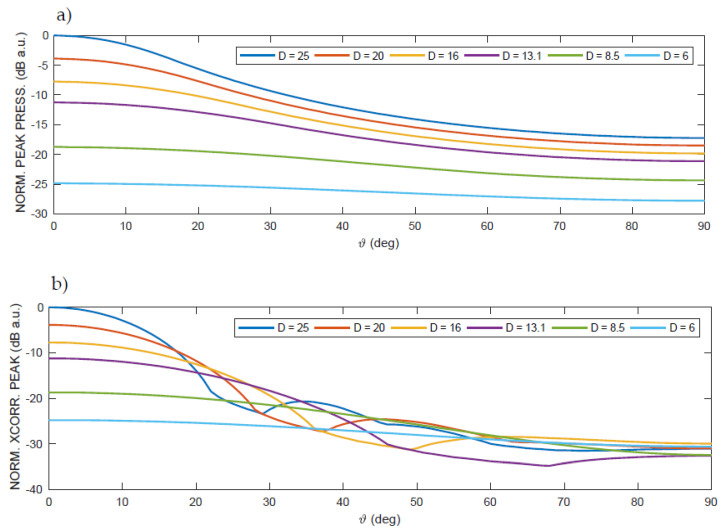

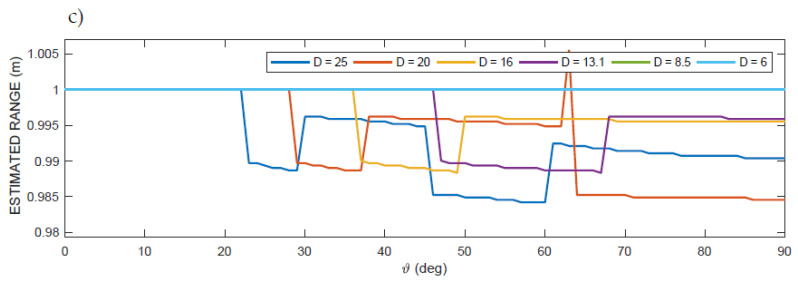
Numerical results at different transducer apertures D = {25, 20, 16, 13.1, 8.5, 6} mm along a semicircular path at distance R = 1 m from the emitting transducer, using a linear chirp with starting frequency f_L_ = 30 kHz and final frequency f_H_ = 50 kHz. A linearized air absorption around 40 kHz (slope 39.3 dB/m·MHz, constant term -0.262 dB/m, i.e., about 0.917 dB/m @ 20 kHz and 1.703 dB/m @ 50 kHz) has been assumed, considering the room temperature 20 °C and atmospheric pressure 1 atm: (**a**) acoustical pressure peak, displayed after normalization and dB conversion, (**b**) cross-correlation peak, displayed after normalization and dB conversion, and (**c**) estimated range from the position of the cross-correlation absolute peak. For the first four aperture diameters, the lag of the cross-correlation peak does not correspond to the correct time of arrival (TOA) (see also, Figure 4).

In Figure 3, it is possible to see the value of the pressure peak, the correlation peak, and the estimated distance when using the position of the correlation peak to evaluate the TOA at varying D within the previously defined set. Given the field symmetry, only the results for angles from 0° (on the axis) to 90° (laterally to the transducer) are shown on a path at a constant distance R = 1 m from the center of the transducer and with varying apertures of D.

Finally, at the bottom of Figure 3, it is possible to see the results of the estimate of the distance R* using the usual technique based on the search for the maximum position of the cross-correlation peak (τ_MAX_) [16,32], which, in favorable conditions, produces the correct estimate of the TOA and, from this, the estimate of the range R*, considering:

(2) $$c_{air}=331.5\sqrt{1+\frac{T}{273.15}},$$

where c_air_ (m/s) is the speed of sound in the air, and T (°C) is the ambient temperature. In particular, the estimate of the range estimate R* is computed as follows:

(3) $$R^{*}=\left(\frac{\tau_{MAX}}{f_{S}}-TOE\right)c_{air}-R_{cal}$$

where τ_MAX_ is the lag of the maximum peak, and *R_cal_* is a calibration constant that takes into account all the fixed delays of the considered system. *R_cal_* is independent of the range, and the time of emission of the ultrasonic signal (TOE) can be assumed known through some a priori operation.

**Figure 4:**
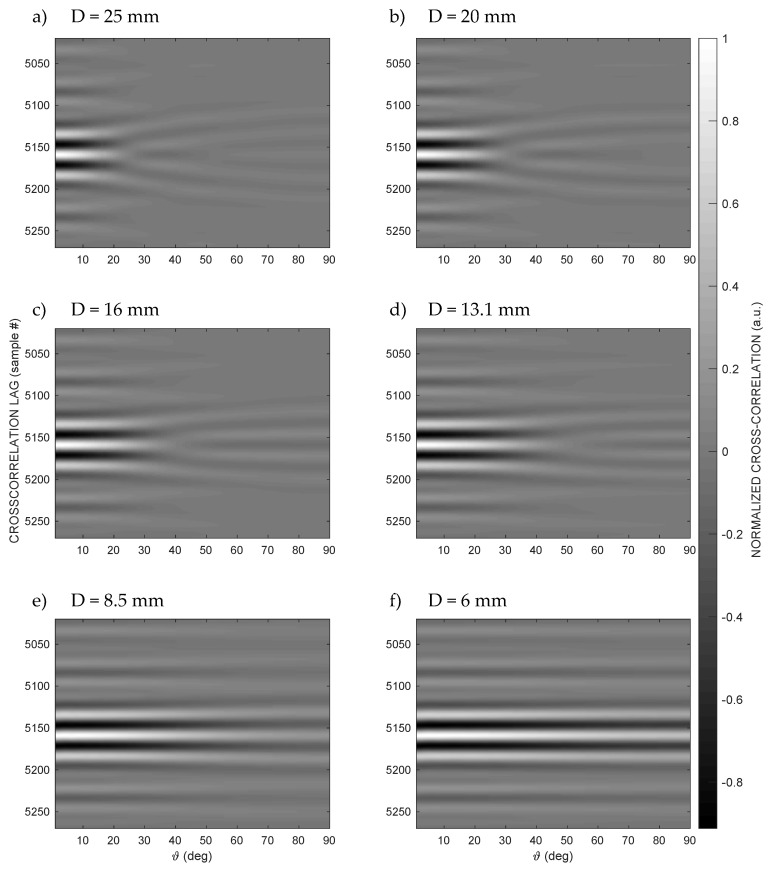
Cross-correlation values along a semicircular path at distance R = 1 m from the emitting transducer at different transducer apertures D = {25, 20, 16, 13.1, 8.5, 6} mm. From D = 25 mm down to D = 6 mm, it is possible to appreciate the progressive appearance of a single correlation peak, which makes the identification of the TOA univocal.

The cross-correlation along the same path considered in Figure 3—that is, along a quarter of a circumference belonging to a plane passing through the emission axis of the transducer with a radius R = 1 m—is shown in Figure 4 as the grayscale amplitude of the cross-correlation with variable lag for each angle ϑ and for the six considered apertures.

**Figure 5:**
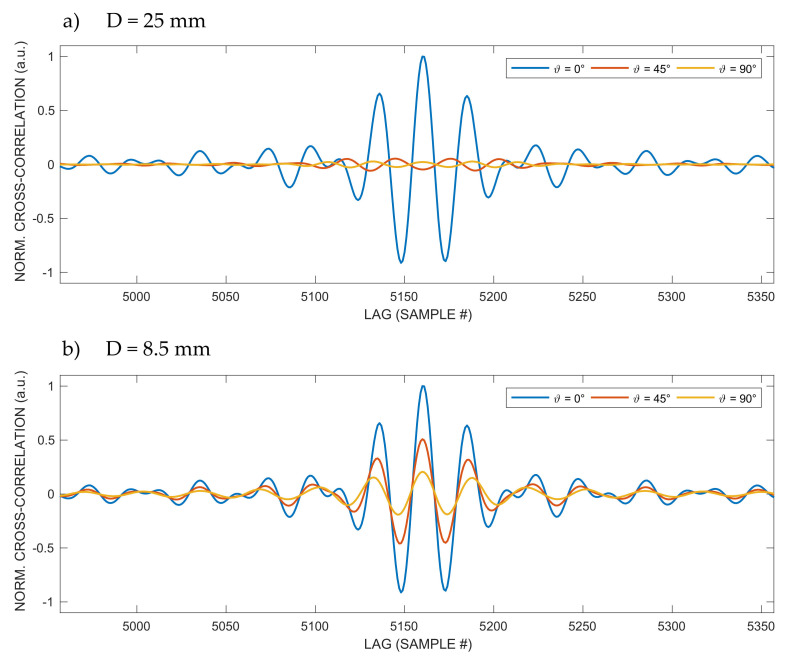
Cross-correlations along a semicircular path at distance R = 1 m from the emitting transducer for two different transducer apertures: (**a**) D = 25 mm and (**b**) D = 8.5 mm. For D = 25 mm, it is possible to see that the single unique peak of the cross-correlation for ϑ = 0° is no longer present at the 45° and 90° angles, while, for D = 8.5 mm, it is possible to appreciate the single correlation peak at all angles. The cross-correlation values are normalized to their maximum value for each aperture: for D = 8.5 mm, the amplitude relative reduction with respect to the increasing angle is much lower than for D = 25 mm, due to the much wider emission of the smaller aperture.

In Figure 5 are displayed the cross-correlations along a semicircular path at a distance R = 1 m from the emitting transducer for two different transducer apertures: D = 25 mm (Figure 5a) and D = 8.5 mm (Figure 5b). The cross-correlation values are normalized to their maximum value for each aperture.

In order to observe in detail the extent and shape of the spatial regions within which it is possible to obtain the typical accuracy of the technique based on the cross-correlation, the ranging error on two rectangular grids of points (see Section A and B of the room volume, Figure 1) was evaluated as a function of D. The grid pitch is 5 cm in the *x* and *z* directions. For each point, the lag of the correlation peak (τ_MAX_) and, from these, the estimates of the distance from the emitter through (3) were obtained. Finally, the competent ground-truth value at each point of the simulation grid was subtracted from the values just obtained, thus generating a grid of estimates of the ranging error.

**Figure 6:**
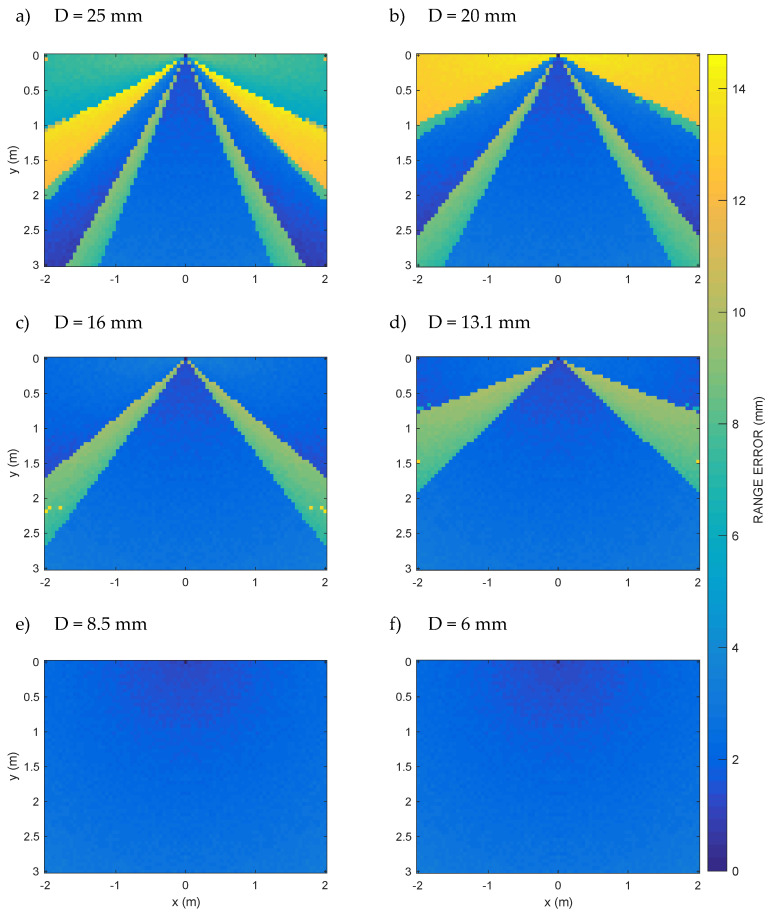
Computed range error at different transducer apertures D = {25, 20, 16, 13.1, 8.5, 6} mm in a dense grid of points (horizontal and vertical step = 0.05 m) belonging to the vertical 4 m × 3 m Section A (see Figure 1); it is possible to appreciate the progressive widening of the cone of the minimum ranging error going from D = 25 mm to D = 6 mm. For aperture values D = 8.5 mm and D = 6 mm, the low error region includes all the half-space in front of the transducer.

Figure 6 shows the ranging error along a rectangular vertical section (Section of the room volume, Figure 1) of 3-m height and 4-m base passing through the center of the transducer, equal to the vertical section of the typical office room taken as a reference in some positioning works [27,33,34], for each aperture D of the set defined above. The grid pitch is 5 cm in the *x* and *y* directions.

**Figure 7:**
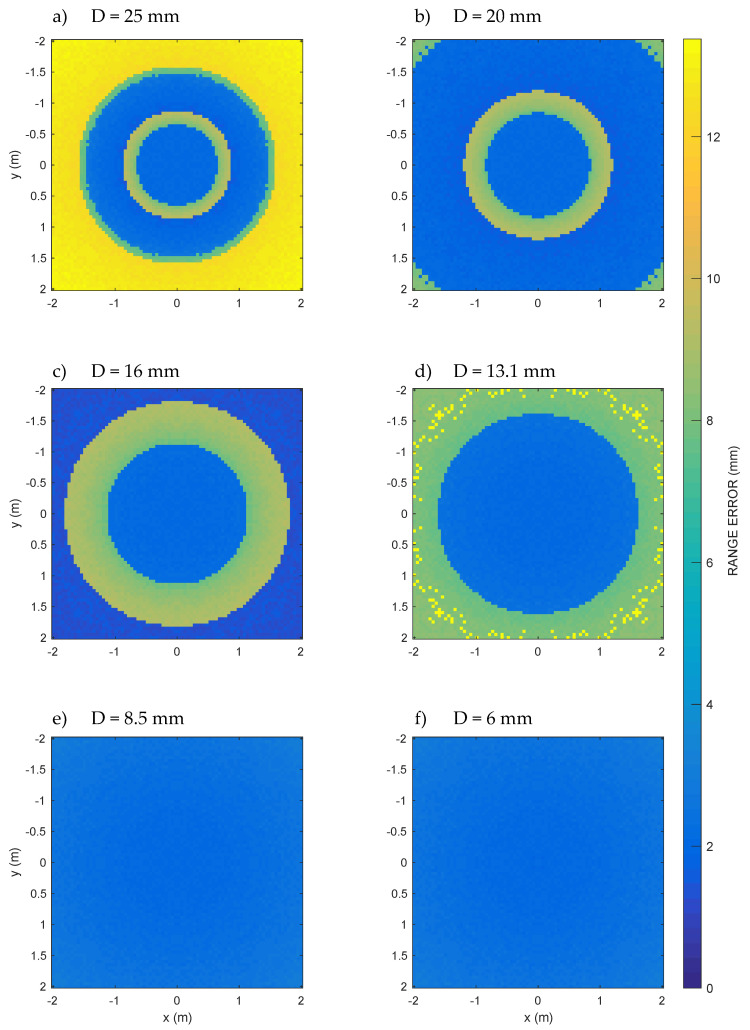
Computed range error at different transducer apertures D = {25, 20, 16, 13.1, 8.5, 6} mm in a dense grid of points (horizontal and vertical step = 0.05 m) belonging to the horizontal 4 m × 4 m Section B (see Figure 1); it is possible to appreciate the progressive widening of the disk of the minimum ranging error going from D = 25 mm to D = 6 mm. For aperture values D = 8.5 mm and D = 6 mm, the low error region includes all the half-space in front of the transducer.

Finally, Figure 7 shows the behavior of the ranging error on a horizontal section of 4 m × 4 m at z = 1.5 m, or halfway between the ground and the ceiling (Figure 1b), for all the apertures considered.

For D from 25 mm to 13.1 mm, Figure 7a–d, it is possible to clearly recognize increasing low error circular areas, which are the circular sections of the low error cones already seen in Figure 6a–d. Using such apertures, therefore, it is not possible to cover the whole room for that height, and things go even worse for high heights, which, however, are certainly of interest for a three-dimensional internal positioning system. On the contrary, the volume of the room is completely covered by the last two apertures D = 8.5 mm and D = 6 mm, as can also be seen from the vertical sections of Figure 6e,f, which show ranging errors everywhere lower than about 3.09 mm. The ranging error is due to the numerical approximations and the sampling frequency chosen for the simulation.

## 4. Discussion

In Figure 3, as expected, the pressure peak drops smoothly and rapidly with the increasing angle ϑ. The behavior of the cross-correlation peak is different, abruptly varying at certain angles. This depends on the shape of the cross-correlation, as shown also in Figure 4.

In general, note that the pressure values are decreasing as the aperture decreases, since the surface power density in emission is kept constant, while the extension of the emitting surface decreases. Furthermore, since the cross-correlation is also proportional to the amplitude of the received signal, its peak value also decreases with the pressure signal. Note that, as the aperture D decreases, the angle up to which the correct estimate is obtained increases; for D = 25 mm, the largest ranging error is obtained, over 15 mm.

Additionally, consider that no noise has been added to show more clearly that the observed phenomenon is due only to the acoustic diffraction that exists regardless of the current SNR level.

In Figure 4, the grayscale amplitude of the cross-correlation with variable lag shows that, up to a certain angle ϑ, the shape of the cross-correlation remains regular, as expected, with only one clearly recognizable peak. In the regions included in the above angles, the correlation-based technique works very well, with errors in the order of the sampling rate of the signal. However, beyond a certain limit angle, the value of which increases as the transducer aperture D decreases, on the other hand, the cross-correlation deforms, with variations in the shape and multiplication of the peaks, with a trend similar to a bifurcation. For angles larger than this limit, it becomes unpractical to identify a peak corresponding to the TOA, simply because it no longer exists; in fact, the peaks of the cross-correlation beyond the limit angle no longer correspond to the correct lag proportional to the TOA, and, therefore, they produce incorrect estimates of the TOA and, as a consequence, of R*. This finally well explains the strange abrupt behavior of the range estimation of Figure 3c.

In Figure 5, it is possible to see that, for D = 25 mm, it is possible to appreciate that the single univocal peak of the cross-correlation for ϑ = 0° is not anymore present at angles 45° and 90°, while, for D = 8.5 mm, it is possible to identify the single correlation peak at all angles. Moreover, for D = 8.5 mm, the amplitude relative reduction with respect to the increasing angle is much lower than the reduction for D = 25 mm, due to the much wider emission of the smaller aperture.

In Figure 6a–d is displayed a zone with a shape similar to a triangle (similar to a cone in three dimensions), with the vertex corresponding to the center of the transducer, inside which the error is minimal, i.e., of the order of the error quantization due to the signal time sampling 1/f_S_. Therefore, let us define this low error triangular region (conical in three dimensions) as that of the correct operation of the ranging system and φ its angle at the vertex. In Table 1, it is possible to see φ and the maximum error as a function of the increasing aperture D values.

Immediately outside this low error area, on the other hand, the abrupt appearance of a higher error, often greater than 11 mm, is observed, which is ultimately produced by the bifurcation of the cross-correlation values, as also shown in Figure 4.

Instead, in Figure 6e,f, no conical region is seen, but a fairly uniform error appears, which is about one order of magnitude lower than that seen in Figure 6a–d. This is the numerical error due to the numerical approximations and the sampling frequency chosen for the simulation, everywhere less than about 3.3 mm. The absence of the low error conical region is due to the fact that, for aperture values D = 8.5 mm and D = 6 mm, the low error region includes all the half-space in front of the transducer.

The isolated points of yellow color (relatively large ranging errors) in Figure 6c,d refer to positions where the peak detection error is large due to the similarity in the height of adjacent peaks of the cross-correlation (see also peaks of almost equal height for ϑ = 45° and ϑ = 90° in Figure 5a). The same phenomenon is also observed in Figure 7d.

In Figure 7a–d, for D from 25 mm to 13.1 mm, it is possible to clearly recognize increasing low error circular areas, which are the circular sections of the low error cones already seen in Figure 6a–d. Using such aperture values, therefore, it is not possible to cover the whole room for that height, and things go even worse for *z* higher than 1.5 m, which, however, is certainly of interest for a three-dimensional internal positioning system. On the contrary, the volume of the room is completely covered by the last two apertures D = 8.5 mm and D = 6 mm, as can also be seen from the vertical sections of Figure 6e,f, which show ranging errors everywhere lower than about 3.09 mm. The ranging error is mainly due to the numerical approximations and the sampling frequency chosen for the simulation.

As a significant result, for apertures D from 25 mm down to 13.1 mm, it is possible to clearly recognize the cone-shaped favorable zone. With these apertures, however, it is not possible to cover the whole room. In fact, the room can only be covered up to a height of less than 1 meter from the floor in the most favorable case. This unfortunately prevents, in many cases, from reaching the coverage required by three-dimensional indoor positioning systems. The room, on the other hand, is completely covered by the last two apertures, as can also be seen from the vertical Section A shown in Figure 6, where, in fact, the conical region is no longer recognized, since the low error area is now extended to the whole volume of the room.

The simulations presented demonstrate that, using Field II in the design phase, by varying the transducer aperture and the others parameters, it is therefore possible to check whether the acoustic coverage required by a specific application is reached, i.e., whether the region of interest for that application is within the region where the ranging error is sufficiently low or not.

## 5. Conclusions

In this paper, Field II, an acoustical simulation software well-established in the field of ultrasound medical imaging, has been applied to the simulation of the acoustic field in air produced by a circular transducer and to the evaluation of a ranging technique based on the measurement of this acoustic field.

The original contribution of this work is to show that Field II can be profitably applied to the problem of ranging with ultrasound in the air. As the first significant result, numerical simulations have shown that it is not enough to guarantee a certain acoustic pressure in a spatial region to reach a certain low level of error. In fact, depending on the angle at which the emitter is seen, the received chirp undergoes a significant aberration in shape compared to that emitted. Shape aberration also occurs to its cross-correlation, so the usual peak detection technique cannot detect the true TOA, regardless of the signal level or SNR.

Field II allows us to observe ranging errors greater than expected in the presence of signal shape aberrations, regardless of the SNR. This means that particular care must be taken in the acoustic design of an ultrasound positioning system and that the use of a numerical simulator such as Field II is necessary in the design phase. With such a tool, in fact, it is possible to evaluate effectively both the acoustic coverage and the accuracy of the ranging technique used.

In particular, it was possible to observe the total coverage of a typical 4 m × 4 m × 3 m room by using a circular aperture of diameter D = 8.5 mm or less, a 30–50-kHz linear chirp signal, and cross-correlation-based peak detection. In this case, the maximum ranging error obtained across the entire volume was about 3.3 mm. Instead, for larger D, outside the favorable regions shown by the numerical simulations, the ranging error increases up to 14.6 mm.

Many applications and services based on ultrasonic positioning systems can benefit from the presented simulation tool.

## Figures and Tables

**Table 1:** Low error cone angle and range maximum error as a function of the aperture D.

Emitter aperture D (mm)	Low Error Cone Vertex Angle φ (°)	Range Maximum Error (mm)
6	180	3.3
8.5	180	3.3
13.1	93.51	13.3
16	73.26	13.7
20	55.84	14.2
25	44.62	14.6
